# A case of incidental renal pelvic basaloid squamous cell carcinoma

**DOI:** 10.1097/MD.0000000000045605

**Published:** 2025-11-14

**Authors:** Zhiqi Li, Tianyun Wang, Yan Wang, Min Su, Zhiyan Ou, Jie Hua, Xiang Kui

**Affiliations:** a Department of Pathology, The Second Affiliated Hospital of Kunming Medical University, China.

**Keywords:** basaloid squamous cell carcinoma, HPV, renal pelvis

## Abstract

**Rationale:**

Basaloid squamous cell carcinoma (BSCC) is a rare subtype of SCC, commonly occurring in the head and neck region, but primary cases in the renal pelvis are extremely rare. This article reports a case of primary BSCC in the renal pelvis.

**Patient concerns:**

A 49-year-old female patient was admitted with nonfunctioning kidneys and a urinary tract infection, and underwent an elective right laparoscopic nephrectomy for the nonfunctioning kidney.

**Diagnoses:**

Renal scan and computed tomography imaging indicated reduced kidney function and hydronephrosis. After clinical evaluation, the diagnosis of nonfunctioning kidney and urinary tract infection was considered.

**Interventions:**

An elective right laparoscopic nephrectomy for the nonfunctioning kidney was performed.

**Outcomes:**

The postoperative pathological diagnosis was renal pelvic BSCC, and the patient received postoperative chemotherapy and is currently followed up for 11 months, with no evidence of tumor recurrence or progression.

**Lessons:**

This patient was admitted with a nonfunctioning kidney, and neither imaging nor clinical assessment considered the possibility of a tumor. However, BSCC is highly malignant and aggressive, warranting attention.

## 1. Introduction

Basaloid squamous cell carcinoma (BSCC) is a rare subtype of squamous cell carcinoma (SCC) that primarily occurs in the head and neck region, although recent reports have indicated its occurrence in various other organs. However, reports of renal pelvis BSCC are extremely rare. Due to its high malignancy and strong invasiveness, and the lack of specificity in clinical symptoms and imaging features, it should be of great concern to clinicians and pathologists. In this report, we present a case of renal pelvis BSCC.

## 2. Case description

### 2.1. General information

A 49-year-old female patient presented to a local hospital in October 2022 with right flank distension and pain. Examination revealed right ureteral stricture accompanied by right renal hydronephrosis, leading to the insertion of a ureteral stent. The patient underwent 2 ureteral stent replacements in November 2022 and March 2023. In June 2023, she was transferred to our hospital for further treatment, and she underwent right ureteral stricture dilation and ureteral stent replacement in June 2023, August 2023, January 2024, and May 2024.

### 2.2. Additional examinations

**Figure 1.**
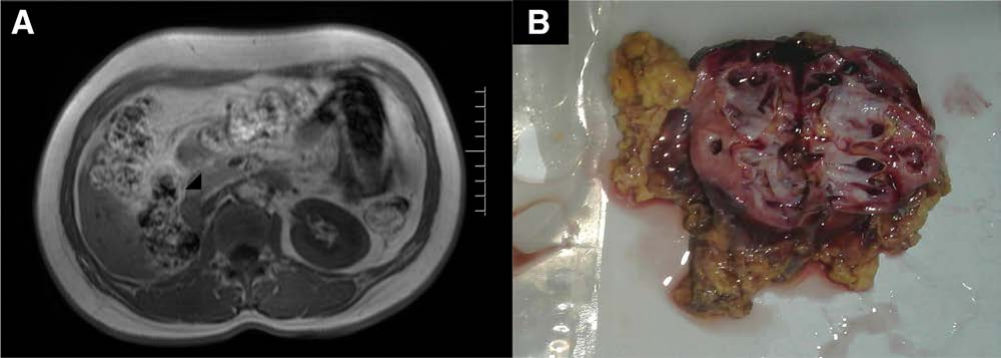
CT and macroscopic. (A) CT shows significant atrophy of the right kidney (arrow). (B) Gross image shows atrophy of the right renal parenchyma, with dilation of the renal pelvis and calyces. CT = computed tomography.

In August 2024, a follow-up renal scan indicated poor blood flow perfusion in the right kidney. Computed tomography examination of the female urinary system revealed a small right kidney with markedly thinned renal cortex, significant hydronephrosis and dilation of the renal pelvis and calyces, and thickened, irregular walls of the right ureter and renal pelvis, suggesting inflammation (Fig. 1A). The patient was admitted with a nonfunctional kidney and urinary tract infection, and was scheduled for a right laparoscopic nephrectomy for the nonfunctional kidney.

### 2.3. Pathological findings

**Figure 2.**
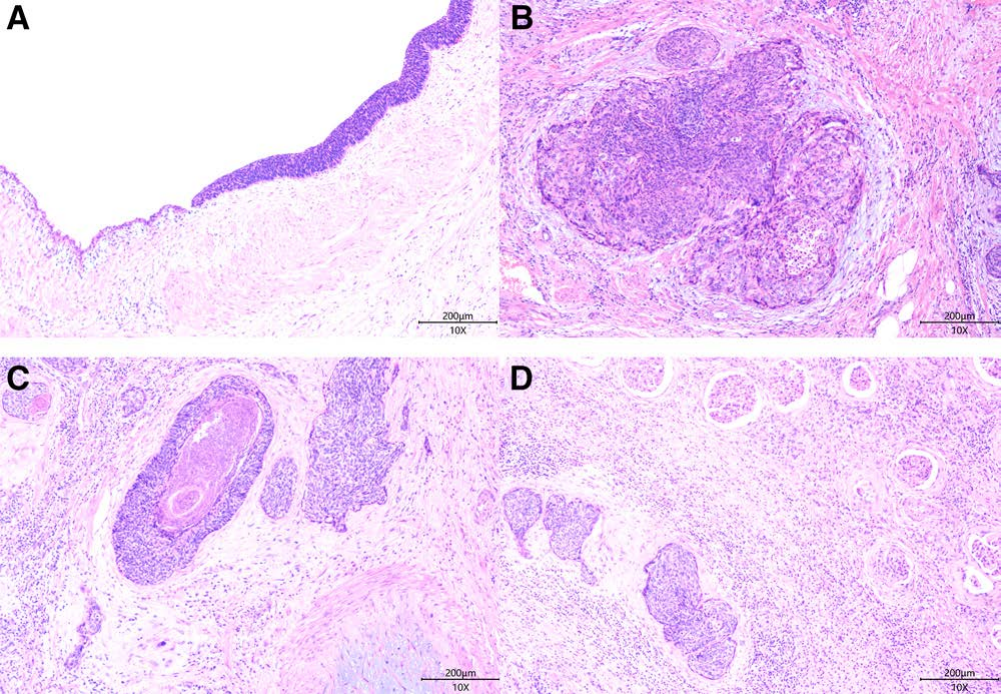
Microscopic features of the lesions. (A) Gradual thickening of the renal pelvic urothelium, transitioning to pleomorphic tumor cells, X100. (B) Tumor cells are arranged in nests of varying sizes, in sheet-like and cord-like structures, with peripheral cells arranged in a palisade pattern, X100. (C) Focal areas of comedonecrosis can be observed at the center of some cancer nests. (D) Renal parenchyma shows tumor involvement, X100.

**Figure 3.**
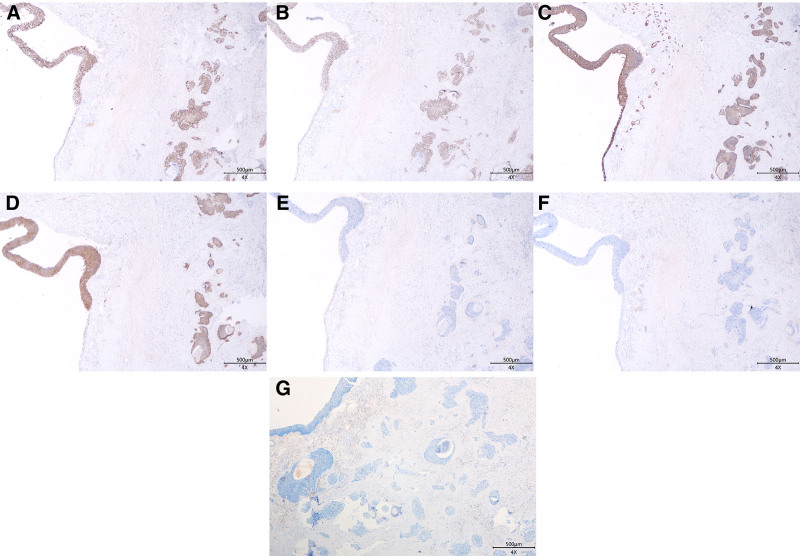
Immunohistochemical results. (A) P40 diffuse nuclear positivity, X40. (B) P63 diffuse nuclear positivity, X40. (C) CK7 diffuse membranous positivity, X40. (D) P16 diffuse membranous positivity, X40. (E) GATA3 positive in urothelial cells, negative in tumor cells, X40. (F) CK20 negative, X40. (G) HPV negative, X40.

Macroscopic: A single right kidney specimen, measuring 8 × 5 × 2 cm, was examined. On cross-section, the renal cortex appears atrophied and thinned, with cystic dilation of the renal pelvis and calyces (Fig. 1B). Microscopic: The renal parenchyma shows signs of atrophy. The urothelial layer of the renal pelvis gradually thickens, progressing into pleomorphic tumor cells (Fig. 2A). These tumor cells are oval or spindle-shaped, with scant cytoplasm, small size, and indistinct nucleoli. Numerous mitotic figures are present (>10/10 HPF). The basal layer is arranged in a trabecular pattern. Multiple discrete infiltrating foci are observed within the renal parenchyma. Tumor cells are organized in nests of various sizes, sheets, and cords, with a trabecular arrangement of cells around the cancer nests (Fig. 2B). Some cancer nests show central punctate necrosis (Fig. 2C). Cancer involvement is also seen in the perirenal fat around the renal pelvis (Fig. 2D), along with nerve invasion. The distal cut edge of the ureter still shows carcinoma in situ. Immunohistochemical results: Tumor cells are diffusely positive for P40, P63, CK7, and P16(Fig. 3A–D), while negative for GATA3, CK20, and HPV (Fig. 3F and G). The KI-67 index is approximately 80%. Pathological diagnosis: BSCC of the renal pelvis, with invasion into the renal parenchyma and perirenal fat tissue. The ureteral surgical margin shows carcinoma in situ of BSCC.

### 2.4. Follow-up treatment and monitoring

**Figure 4.**
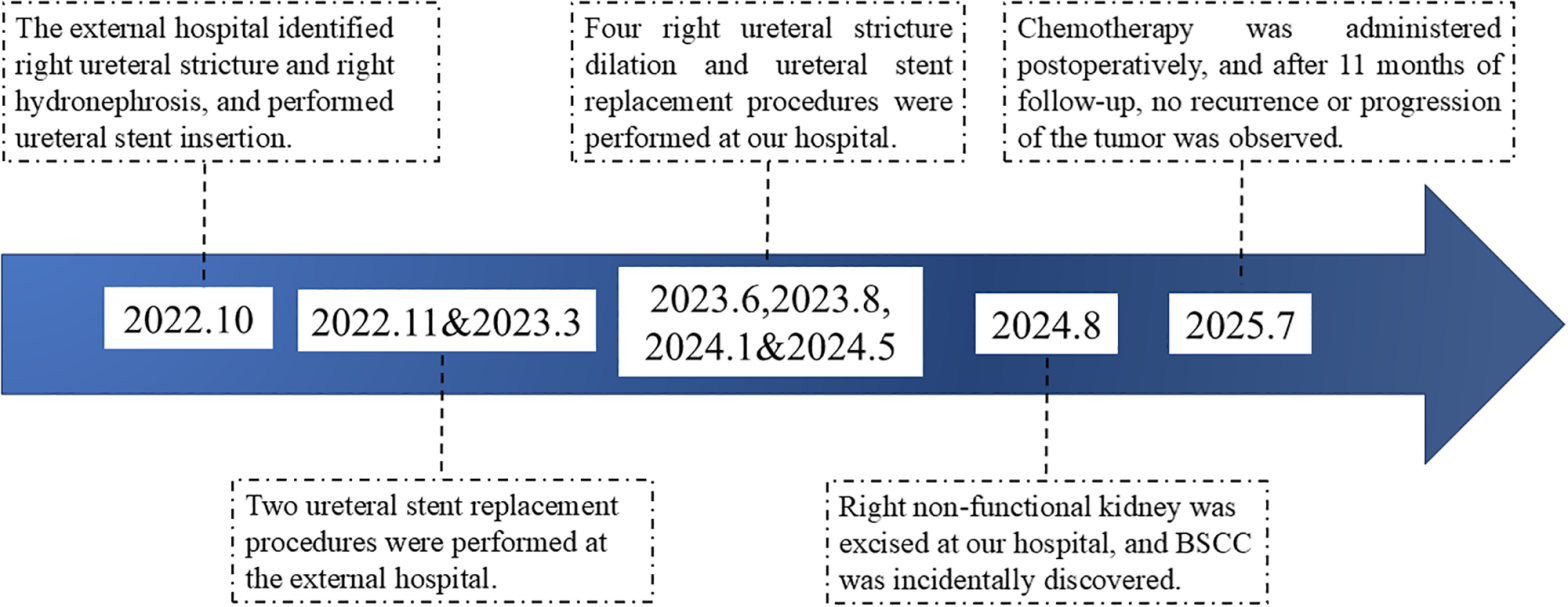
Timeline of illness.

Following a diagnosis of BSCC, the patient received adjuvant chemotherapy with gemcitabine and cisplatin. By the 11-month follow-up, no tumor progression or recurrence had been detected. Figure 4 depicts the clinical course from the initial presentation with back pain in October 2022, when treatment was initiated, to the latest follow-up in July 2025.

## 3. Discussion

The term “BSCC” was first introduced by Wain et al in 1986 to describe a subtype of SCC, primarily occurring in the head and neck region. Primary urothelial BSCC is rare, with only 6 reported cases to date, including 1 in the renal pelvis, 1 in the ureter, and 4 in the bladder.^[1–6]^ This case represents the second reported instance of BSCC in the renal pelvis. The morphology and immunohistochemistry of BSCC are similar to those of primary tumors in the head and neck region.^[7]^ The tumor cells consist of densely arranged basaloid-type cell nests with scant cytoplasm. The tumor nests have well-defined borders, and central necrosis is observed in some of the nests. Tumor cells typically show diffuse strong positivity for P40, P63, CK5/6, and CK7, while GATA3, Villin, and CK20 are negative. The tumor cells consist of densely arranged basaloid-type cell nests with scant cytoplasm. The tumor nests have well-defined borders, and central necrosis is observed in some of the nests. Tumor cells typically show diffuse strong positivity for P40, P63, CK5/6, and CK7, while GATA3, Villin, and CK20 are negative. This patient has a history of hydronephrosis and 20 months of ureteral stent placement. It is speculated that the recurrent friction of the catheter on the urothelium, combined with prolonged chronic inflammation, may have led to urothelial and squamous metaplasia, which subsequently progressed to carcinogenesis and developed into BSCC. Based on these observations, we propose that long-term medical implants (e.g., ureteral stents) placed in anatomically vulnerable areas such as the renal pelvis, which are prone to chronic inflammation and secondary tumors, could be a potential risk factor for the development of these rare cancers. Therefore, we highly recommend increased clinical follow-up and imaging monitoring for patients with long-term indwelling urinary devices, to facilitate early detection and timely intervention.

Some researchers currently believe that the development of BSCC in most areas is linked to HPV infection, including in urothelial BSCC.^[6]^ Of the 6 previously reported cases, 3 tested positive for HPV by in situ hybridization, while the remaining 3 did not provide detailed information. The mechanism of HPV infection involves the HPV oncoprotein E7 inactivating the retinoblastoma protein, leading to the loss of transcriptional inhibition of p16 and its subsequent overexpression.^[8]^ In cervical cancer, strong positivity for p16 is closely associated with HPV infection. However, some studies suggest that in urothelial epithelium, the degree of immunohistochemical positivity for p16 is not linked to HPV infection, and p16 negativity does not rule out HPV infection.^[9]^ In this case, although there was diffuse strong positivity for p16, further HPV immunohistochemistry was negative (Fig. 3G), indicating that the urothelial BSCC in this patient is not related to HPV infection. Although several reports suggest a possible link between HPV infection and the development of BSCC, no HPV infection was detected in the cases studied here, suggesting that this link may not be universally applicable. Additionally, while p16 immunohistochemistry positivity is often used as a surrogate marker for HPV infection in some tumor types (e.g., cervical cancer), p16 expression in urothelial tumors may not be correlated with HPV infection. Therefore, more specific detection techniques, such as in situ hybridization or next-generation sequencing, are recommended to provide more direct evidence of HPV infection.

Currently, clinical data on renal pelvic BSCC are limited. As a result, treatment and prognosis evaluation mainly rely on data related to renal pelvic SCC. Because renal pelvic BSCC has nonspecific symptoms, patients are often diagnosed only when the disease has reached an advanced stage or has spread. At present, there is no standard treatment protocol. Surgery is considered the primary treatment for renal pelvic BSCC. Regarding prognosis, studies show that the 5-year survival rate for renal pelvic SCC is only 7.7%, with a median survival time of 7 to 10 months. In studies of head and neck BSCC, the average follow-up time suggests that tumor survival rates are generally lower than those for SCC, and the rate of distant metastasis is higher than that for SCC.^[10]^ The patient received standard chemotherapy after surgery and is currently being followed up for 11 months, with no signs of tumor recurrence or progression. Therefore, comprehensive surgery followed by chemotherapy can benefit patients with BSCC. Based on this, we can infer that the prognosis for renal pelvic BSCC may be even worse. This case suggests that aggressive multimodal therapy, including radical surgery and adjuvant chemotherapy, could improve the prognosis of BSCC. Given that this patient has carcinoma in situ involving the ureteral stump, there is a possibility that carcinoma in situ could progress further to the bladder and urethra. We recommend regular cystoscopic monitoring and long-term follow-up for these patients to facilitate early detection of potential recurrence or progression. Additionally, in addition to conventional radiotherapy and chemotherapy, immunotherapy has emerged as an effective antitumor treatment and is being used in the management of various malignancies. However, despite showing significant efficacy in tumor destruction, immunotherapy still faces challenges, including low overall response rates, susceptibility to acquired resistance, and treatment-related adverse events.^[11,12]^ Currently, the efficacy of immunotherapy in BSCC is still unclear. Particularly in advanced patients unresponsive to conventional treatments, immunotherapy may offer unexpected benefits; however, further research is needed to validate this.

Finally, this case further contributes to the clinical evidence supporting BSCC as a distinct pathological entity, offering detailed insights into its disease progression, treatment response, and prognostic features. In clinical practice, rare tumors like BSCC, which present diagnostic challenges, should be given high priority. Future research should explore molecular mechanisms in greater depth to better elucidate its pathogenesis and provide a theoretical basis for improving patient outcomes.

## 4. Conclusions

Renal pelvic BSCC is relatively rare in clinical practice, with most patients having a history of long-term kidney stones, hydronephrosis, or previous catheter placement. The symptoms of the patient lack specificity, and early radiological diagnosis is also challenging, often being confused with inflammation. In this case, the patient was admitted with nonfunctioning kidneys, and neither imaging nor clinical assessment considered the possibility of a tumor. During surgery, the resection of the ureteral stump was performed too close to the tumor, resulting in the presence of carcinoma in situ at the surgical margin, which may impact further postoperative treatment. Therefore, for patients with long-term urothelial friction and chronic urinary tract infections, clinical and imaging exams should be careful to detect urothelial SCC, especially BSCC. Pathologists should consider both urothelial SCC and BSCC in the differential diagnosis and ensure proper tissue sampling. Currently, there is limited clinical data on urothelial BSCC, and there is no clear guidance for treatment and prognosis. The role of HPV in the development of BSCC in the urinary tract and whether strong P16 positivity is linked to HPV infection requires further study and more case reports.

## Acknowledgments

We would like to express our sincere gratitude to the patient for her participation in this study and for providing consent to publish her case.

## Author contributions

**Conceptualization:** Zhiqi Li, Min Su, Jie Hua.

**Supervision:** Yan Wang.

**Writing – review & editing:** Tianyun Wang, Zhiyan Ou, Xiang Kui.
